# Quantitative ultrasound techniques and biochemical markers to assess liver steatosis and fibrosis in newly diagnosed acromegaly

**DOI:** 10.1007/s40618-024-02384-5

**Published:** 2024-05-06

**Authors:** M. Coskun, H. N. Sendur, A. Babayeva, M. N. Cerit, E. T. Cerit, M. M. Yalcin, A. E. Altinova, M. Akturk, M. A. Karakoc, F. B. Toruner

**Affiliations:** 1 Department of Endocrinology and Metabolism, Faculty of Medicine, Gazi University, Ankara, Turkey; 2 Department of Radiology, Faculty of Medicine, Gazi University, 06100 Ankara, Turkey

**Keywords:** Acromegaly, CK18, IGFBP7, SWE, Liver steatosis, Liver stiffness

## Abstract

**Purpose:**

The liver is known to be protected from steatosis under the influence of high GH/IGF-1. Cytokeratin 18 (CK18) and insulin-like growth factor binding protein 7 (IGFBP7) increase in liver steatosis and fibrosis. The aim of this study was to use quantitative ultrasound techniques and biochemical markers to assess liver steatosis and liver fibrosis in newly diagnosed acromegaly.

**Methods:**

This single-center, cross-sectional study included 23 patients with newly diagnosed acromegaly and 46 age, sex, body mass index (BMI) and waist circumference (WC)-matched controls. Liver steatosis was assessed using tissue attenuation imaging (TAI), and stiffness, indicative of fibrosis, was assessed by shear wave elastography (SWE). Serum IGFBP7 and CK18 were studied by ELISA.

**Results:**

The acromegaly group had significantly lower liver steatosis (p = 0.006) and higher liver stiffness (p = 0.004), serum IGFBP7 (p = 0.048) and CK18 (p = 0.005) levels than the control group. The presence of fibrosis (p = 0.012) was significantly higher in the acromegaly group than in the control group. Moreover, CK18 was positively correlated with liver stiffness, WC, HOMA-IR, HbA1c, and triglyceride. In the acromegaly group, liver steatosis was negatively correlated with GH level. Stepwise multiple linear regression analysis revealed that BMI (p = 0.008) and CK18 (p = 0.015) were independent risk factors for increased liver stiffness.

**Conclusion:**

This study showed that there was an increased presence of liver fibrosis independent of liver steatosis in newly diagnosed acromegaly. Serum CK18 appears to be a potential marker of increased liver fibrosis in acromegaly.

## Introduction

Acromegaly is a disease characterized by excessive secretion of growth hormone (GH) and insulin-like growth factor-1 (IGF-1) [1]. While increased GH in acromegaly causes a decrease in total body fat by increasing lipolysis and lipid oxidation, it is known that ectopic adiposity, particularly intramuscular adiposity, increases due to the effect of insulin resistance [2, 3]. Contrary to expectations, however, a limited number of studies have shown that ectopic fat deposition, but not hepatic adiposity, increases in patients with acromegaly. On the other hand, it has been reported that hepatic adiposity increases after the control of the disease [3, 4]. In addition, conflicting results were found in a limited number of studies in which liver stiffness was evaluated [5, 6].

Non-alcoholic fatty liver disease (NAFLD) is accepted as the hepatic manifestation of metabolic syndrome and is closely linked to obesity, insulin resistance (IR), type 2 diabetes (T2D), hypertension (HT), and dyslipidemia [7]. Metabolically, reduced GH axis activity in individuals has been associated with increased intrahepatic lipid accumulation [8]. Generally, NAFLD is classically defined as a progressive disease and includes a wide range of clinical conditions, from simple steatosis to nonalcoholic steatohepatitis (NASH) which is associated with inflammation, fibrosis and has potential to progress to liver cirrhosis [9].

Due to the difficulties associated with liver biopsy, which is the gold standard for diagnosing NAFLD, there is growing interest in non-invasive methods such as imaging and serum biomarkers for assessing steatosis and fibrosis [10, 11]. Recently, quantitative ultrasonography (QUS) techniques derived from radiofrequency data analysis have been used as non-invasive and objective tools for assessing fatty liver disease. The Tissue Attenuation Imaging (TAI) technique can be used to detect and grade hepatic steatosis by measuring Attenuation Coefficient (AC) values [12]. Shear wave elastography (SWE) measurements can be used to assess fibrosis [13]. On the other hand, IGF binding protein 7 (IGFBP7), also known as IGFBP-related protein 1, is thought to be associated with the presence of insulin resistance and correlates closely with the degree of steatosis and fibrosis in patients with NAFLD [14]. Studies have demonstrated a correlation between increased IGFBP7 levels and the severity of steatosis and fibrosis in patients with NAFLD [15, 16]. Conversely, a decrease in IGFBP7 has been shown to slow the progression of NAFLD and prevent fibrosis [17]. Cytokeratin (CK18) is a marker of cell apoptosis, and its increase is considered an indicator of increased steatohepatitis and liver fibrosis [18]. CK18 is a reliable indicator of the conversion from biopsy-proven fatty liver to steatohepatitis and the presence of mild fibrosis in the liver [18–20]. CK18 can be used as a non-invasive marker to show mild fibrosis that cannot be detected by the Fibrosis-4 (FIB-4) score, considered a strong indicator for fibrosis [21].

Although the effects of serum GH/IGF-1 levels on hepatic steatosis are known, there is limited data on hepatic steatosis and hepatic fibrosis in patients with newly diagnosed acromegaly. To the best of our knowledge, no studies in the literature have demonstrated a relationship between CK18, IGFBP7, and liver steatosis or liver fibrosis in newly diagnosed acromegaly. The aim of this study was to investigate liver steatosis and the potential for liver fibrosis development in patients with newly diagnosed acromegaly, using QUS techniques (TAI and SWE) and biochemical markers (CK18 and IGFBP7).

## Materials and methods

### Study design and subjects

The study was designed as a cross-sectional, single-center, matched case–control study. Acromegaly was diagnosed by failure of suppression of serum GH concentrations below 1 ng/mL after a 75-g oral glucose tolerance test (OGTT) together with fasting serum IGF-1 levels above the normal ranges for age and gender, and the presence of clinical features. The IGF-1 upper limit of normal (IGF-1 ULN) was calculated by dividing IGF-1 by the upper limit of IGF-1 based on age and gender [22]. Consecutive patients with newly diagnosed acromegaly admitted to our clinic were assessed for inclusion in the study. Two patients were excluded due to pre-existing conditions: one had chronic liver disease, and the other was taking insulin for poorly controlled diabetes. The control group consisted of individuals who attended our outpatient clinic for routine check-up. For each case, two age, sex, body mass index (BMI) and waist circumference (WC)-matched controls were selected.

Exclusion criteria for the selection of the patient and control groups were to be older than 65 years or younger than 18 years old and to have excess alcohol consumption, a history of toxic, autoimmune, viral or metabolic liver disease, secondary causes of fatty liver (e.g., use of systemic steroids, tamoxifen and methotrexate), chronic liver disease of any cause, cirrhosis, any malignancy, chronic renal failure, chronic respiratory failure and cardiac disease. Control subjects known to have NAFLD and diabetes were not included***.*** The study only included patients with newly diagnosed acromegaly to eliminate any potential treatment effects. Since acromegaly often leads to diabetes due to increased insulin resistance, we only included patients with controlled diabetes (glycosylated haemoglobin (HbA1c) levels < 7%) who were not using insulin.

### Physical examination and body composition analysis

Blood pressures and mean arterial pressures were measured. Bioelectrical impedance analysis (BIA) was performed using the Tanita BC-418 MA Body Composition Analyzer (TANITA Corp., Tokyo, Japan). Height, weight, body mass index (BMI), WC, total body fat mass and percentage were all recorded.

### Biochemical analyzes

Serum samples for analysis were obtained early in the morning after an overnight fast. Serum fasting glucose, insulin, total cholesterol (Tchol), high-density lipoprotein (HDL) cholesterol, low-density lipoprotein (LDL) cholesterol, and triglyceride (TG) levels, HbA1c, Alanine aminotransferase (ALT), Aspartate aminotransferase (AST), Gamma glutamyltransferase (GGT), Alkaline phosphatase (ALP) of the patient group and the control group, in addition to the GH and IGF-1 values of the patient group, were recorded. The Homeostatic Model Assessment for Insulin Resistance (HOMA-IR) was calculated using the (glucose (mg/dL) x insulin (µIU/mL) /405) formula.

For CK18 and IGFBP7 measurements, patient and control samples were centrifuged and stored at −80 °C until the day of analysis. Before analysis, serum samples were first transferred to −20 °C and then to room temperature. Elabscience Human CK18 ELISA kit (detection range 6.25–400 mIU/mL, sensitivity < 3.75 mIU/ml, coefficient of variation (CV) < 10%) and Elabscience Human IGFBP7 ELISA (detection range 0.94–60 ng/ml, sensitivity < 0.56 ng/ml, CV < 10%) were used. The kits were used following the manufacturer's instructions, applying a 1:3 dilution to the serum samples.

The presence of any three of the five criteria was defined as metabolic syndrome (MS); elevated WC (≥ 102 cm in men or ≥ 88 cm in women), elevated TG (≥ 150 mg/dL), reduced HDL (< 40 mg/dL in men, < 50 mg/dL in women) elevated blood pressure (BP) (≥ 130 mm Hg systolic BP or ≥ 85 mm Hg diastolic BP), and elevated fasting glucose (≥ 100 mg/dL) [23].

### Liver ultrasound

Ultrasound examinations were performed by two radiologists, each with over 10 years of experience in abdominal radiology, who were blinded to the patients’ clinical status. All patients had fasted for at least 6 h before undergoing ultrasound examinations using a single ultrasound machine (RS85 Prestige, Samsung Medison Co. Ltd) equipped with a convex transducer (1–7 MHz). The imaging of patients was performed in the supine position with the right intercostal approach and the patient’s right hand positioned over the head to increase the width of the intercostal spaces. The radiologist, at first, examined the liver along its course and then measured the liver length in the midclavicular line in centimetres (cm), while the patient was in the supine position. Secondly, stiffness measurements of the liver using SWE imaging were obtained in accordance with the Society of Radiologists in Ultrasound (SRU) guideline [24]. Five consecutive stiffness measurements with breath-hold situation during neutral breathing. The radiologists paid particular attention to avoid applying any pressure with the transducer during the SWE imaging. One cm diameter circular regions of interest are used to measure the liver stiffness values. According to the manufacturer’s recommendations, the appropriate Reliability Measurement Index (RMI) value (> 0.4) is used as a quality indicator for stiffness measurements. Patients with increased liver stiffness (≥ 5kPa) and increased fibrosis (8 > kPa) were noted [13, 24]. The median value of five measurements in kilopascals (kPa) was noted for analyses. The stiffness measurements were considered as reliable if the interquartile range to median value was lower than 30%.

Lastly, the hepatic fat content was quantified by AC measurements using the TAI technique. The AC values were reported in units of dB/cm/MHz and only the values with R^2^ ≥ 0.6 were considered reliable. Five measurements at mid-breath hold were obtained for AC and the median of the measurements was noted. The cut-off value of 0.75 dB/cm/MHz, found in comparison with Magnetic Resonance Imaging Proton Density Fat Fraction (MRI-PDFF) for predicting increased hepatic steatosis (> 5%) with TAI technique, was accepted as the cut-off value for hepatic steatosis [25]. Cases with increased hepatic steatosis detected by TAI technique (AC > 0.75 dB/cm/MHz) were classified as NAFLD.

### Ethical considerations

The study protocol was approved by Local Ethics Committee (Date: 27.06.22, No: 520). Written informed consent was obtained from all participants.

### Statistical analysis

When determining the number of individuals, it was planned to include each case and two controls per case due to the high prevalence of NAFLD in the community. Based on similar studies, when the effect power was determined to be 1.63 using Cohen’s method, 95% power, and a Type I error (alpha) of 0.05 in the analysis performed with the G-Power 3.1.9.4 program, it was determined that at least 8 and 16 individuals were required for the acromegaly and control groups respectively, much lower than the actual study.

Commercial statistical software, Statistical Package for the Social Sciences (SPSS) version 22.0 (IBM Corp., Armonk, NY, USA) was used for statistical analyses. The Shapiro–Wilk test was used to assess the conformity of continuous variables to normal distribution, while the homogeneity of variance was evaluated using Levene’s test. Continuous variables with a normal distribution were presented as the mean ± standard deviation (SD). Continuous variables that were not normally distributed were presented as the median and interquartile range (25-75th percentile). Student’s t-test was used for normally distributed continuous data and Mann—Whitney U test for not-normally distributed data.

The relationships between variables that did not provide the assumption of normality were evaluated using Spearman’s Rho correlation coefficient while the relations between variables that provided the normality assumption were evaluated with the Pearson correlation coefficient.

The effects of BMI, HOMA-IR, HDL cholesterol, TG, CK18, and IGFBP7 variables, which are thought to be associated with liver stiffness and steatosis, were planned to be evaluated using a stepwise multiple linear regression analysis. To ensure the assumption of normality, liver stiffness measurement was included in the model with an inverse transformation. Linear regression analysis could not be performed for the TAI variable due to its lack of normality. Due to the high correlation coefficient between glucose, HOMA-IR and HbA1c, HOMA-IR was included in the model.

The error rate (α = 0.05) was determined in all tests, and the difference between the groups was considered statistically significant when p < 0.05.

## Results

**Fig. 1:**
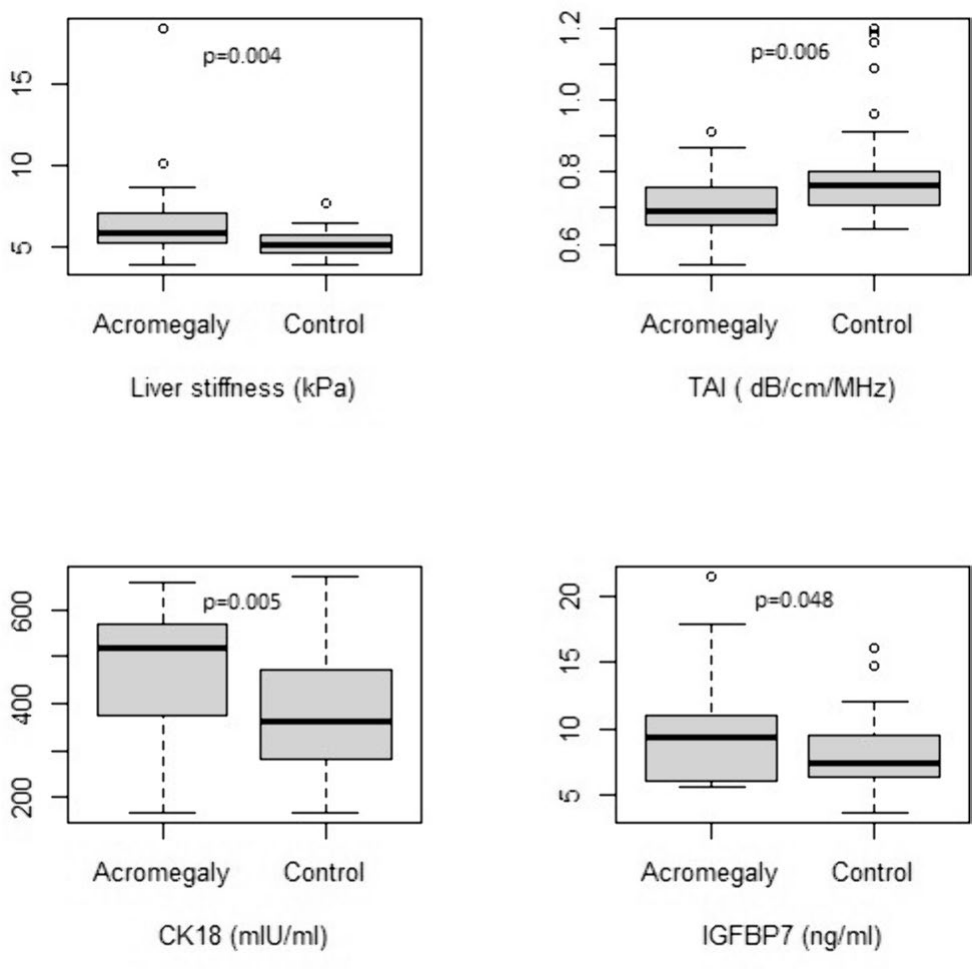
The results of serum CK18, IGFBP7 and liver quantitative ultrasound

Twenty-three patients with acromegaly and 46 controls were included in the study. In the acromegaly group, the median (25–75th percentile) age was 42 (32–51) years, and 60.9% (n = 14) were female. In this group, 34.8% (n = 8) had diabetes, 30.4% (n = 7) had hypertension, and none of the patients had hormone deficiencies. Six patients with diabetes were diagnosed simultaneously with acromegaly, and two were diagnosed within 2 years preceding the diagnosis of acromegaly. As shown in Table 1, while the acromegaly and control groups exhibited similarities in terms of age (p = 0.848), sex (p = 1.0), presence of metabolic syndrome (p = 0.108) WC (p = 0.474), BMI (p = 0.588), and body fat mass (p = 0.105), the acromegaly group had a higher mean arterial pressure (p = 0.002) and lower body fat percentile (p = 0.040) than the control group. The acromegaly group had higher levels of fasting glucose (p = 0.001), insulin (p = 0.003), HOMA-IR (p < 0.001), HbA1c (p < 0.001) and ALP ( p = 0.002) than the control group, while the two groups had similar AST (p = 0.637), ALT (p = 0.575), GGT (p = 0.369), Tchol (p = 0.470), LDL cholesterol (p = 0.529), HDL cholesterol (p = 0.476) and TG (p = 0.633). Additionally, IGFBP7 (p = 0.048) and CK18 (p = 0.005) were higher in the acromegaly group than the control group (Fig. 1). According to the QUS measurement, the liver length measurements were similar between the acromegaly and control group (p = 0.150). However, liver stiffness (p = 0.004), presence of increased liver stiffness (p = 0.014), and presence of increased fibrosis (p = 0.012) were significantly higher in the acromegaly group compared to the control group. Additionally, the liver TAI (p = 0.006) and presence of NAFLD (p = 0.039) were lower in the acromegaly group than the control group (Fig. 1).

**Table 1:** Clinical, laboratory and ultrasonographic characteristics of the individuals

	Acromegaly (23)	Control (46)	p value
Age, years	42.0 (32–51)	39.5 (30–52)	0.848
Gender, Female, % (n)	60.9 (14)	60.9 (28)	1
Diabetes mellitus, % (n)	34.8 (8)	–	–
Hypertension, % (n)	30.4 (7)	–	–
Metabolic syndrome, % (n)	47.8 (11)	28.3 (13)	0.108
Mean arterial pressure, mmHg	96.3 (83.33–98.33)	86.7 (76.67–93.33)	**0.002**
Waist circumference, cm	96.5 ± 14.4	98.9 ± 12.1	0.474
BMI, kg/m^2^	27.3 (26.7 – 30.0)	28 (25.8–30.6)	0.588
Body fat, kg	19.91 ± 8.82	23.32 ± 7.76	0.105
Body Fat, %	24.68 ± 8.95	29.16 ± 8.08	**0.040**
GH, ng/mL	8.7 (3.53–24)	–	–
IGF-1, ULN	2.4 (1.75–3.68)	–	–
Fasting glucose, mg/dL	101 (85–119)	85 (80–90)	**0.001**
Insulin, µIU/mL	14 (7.6–22)	7.8 (5.9–11.13)	**0.003**
HOMA-IR	3.32 (1.80–6.58)	1.64 (1.16–2.52)	** < 0.001**
HbA1c, %	6.2 ± 0.6	5.5 ± 0.4	< **0.001**
AST, U/L	19 (15–26)	20 (17–23)	0.637
ALT, U/L	16 (13–22)	17 (14–23)	0.575
ALP, U/L	99 (80–118)	71.5 (57–88)	**0.002**
GGT, U/L	15 (12–21)	18 (13–27)	0.369
TChol, mg/dL	186.7 ± 36.8	193.2 ± 34.1	0.470
LDL cholesterol, mg/dL	112.3 ± 29.2	117.2 ± 30.6	0.529
HDL cholesterol, mg/dL	47.3 (37.6–58)	49 (38–59)	0.476
TG, mg/dl	129 (57–195)	102.5 (72–166)	0.633
IGFBP7, ng/ml	9.4 (6.08–11.09)	7.5 (6.37–9.51)	**0.048**
CK18, mIU/mL	469.4 ± 134.5	371.1 ± 131.8	**0.005**
Liver length, cm	14.0 ± 2.0	13.3 ± 1.6	0.150
Liver stiffness, kPa	5.8 (5.2–7.2)	5.1 (4.6–5.7)	**0.004**
Increased liver stiffness, % (n)	82.6 (19)	52.2 (24)	**0.014**
Increased liver fibrosis, % (n)	13 (3)	0	**0.012**
TAI, dB/cm/MHz	0.69 (0.65–0.76)	0.76 (0.71–0.81)	**0.006**
NAFLD, % (n)	26.1 (6)	52.2 (24)	**0.039**

Data were presented as Mean ± SD or Median (25–75th percentile). Pearson’s χ2 (chi-square) test method for categorical, Student’s t-test for normally distributed continuous data and Mann—Whitney U test for not normally distributed data were applied

*BMI* body mass index, *GH* growth hormone, *IGF-1* insulin-like growth factor-1, *Tchol* total cholesterol, *TG* triglyceride, *HDL* high-density lipoprotein cholesterol, *LDL* low-density lipoprotein cholesterol, *HOMA-IR* Homeostatic Model Assessment for Insulin Resistance, *HbA1c* glycosylated hemoglobin, *ALT* Alanine aminotransferase, *AST* Aspartate aminotransferase, *GGT* Gamma glutamyltransferase, *ALP* Alkaline phosphatase, *TAI* Tissue attenuation imaging, *NAFLD* Non-alcoholic fatty liver disease, *IGFBP7* Insulin like growth factor binding protein 7, *CK18* cytokeratin 18

Statistically significant p values were given in bold

**Fig. 2:**
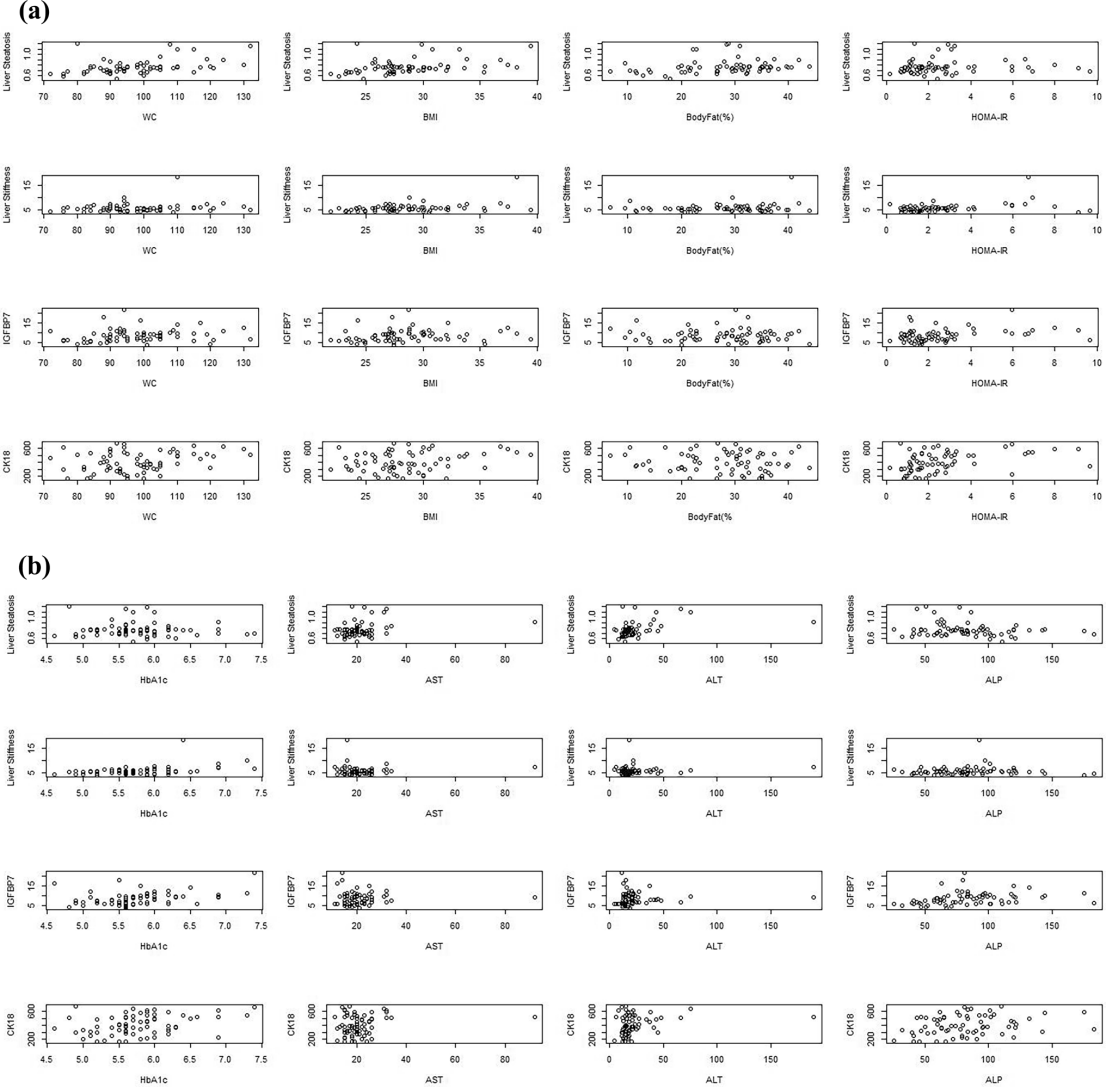

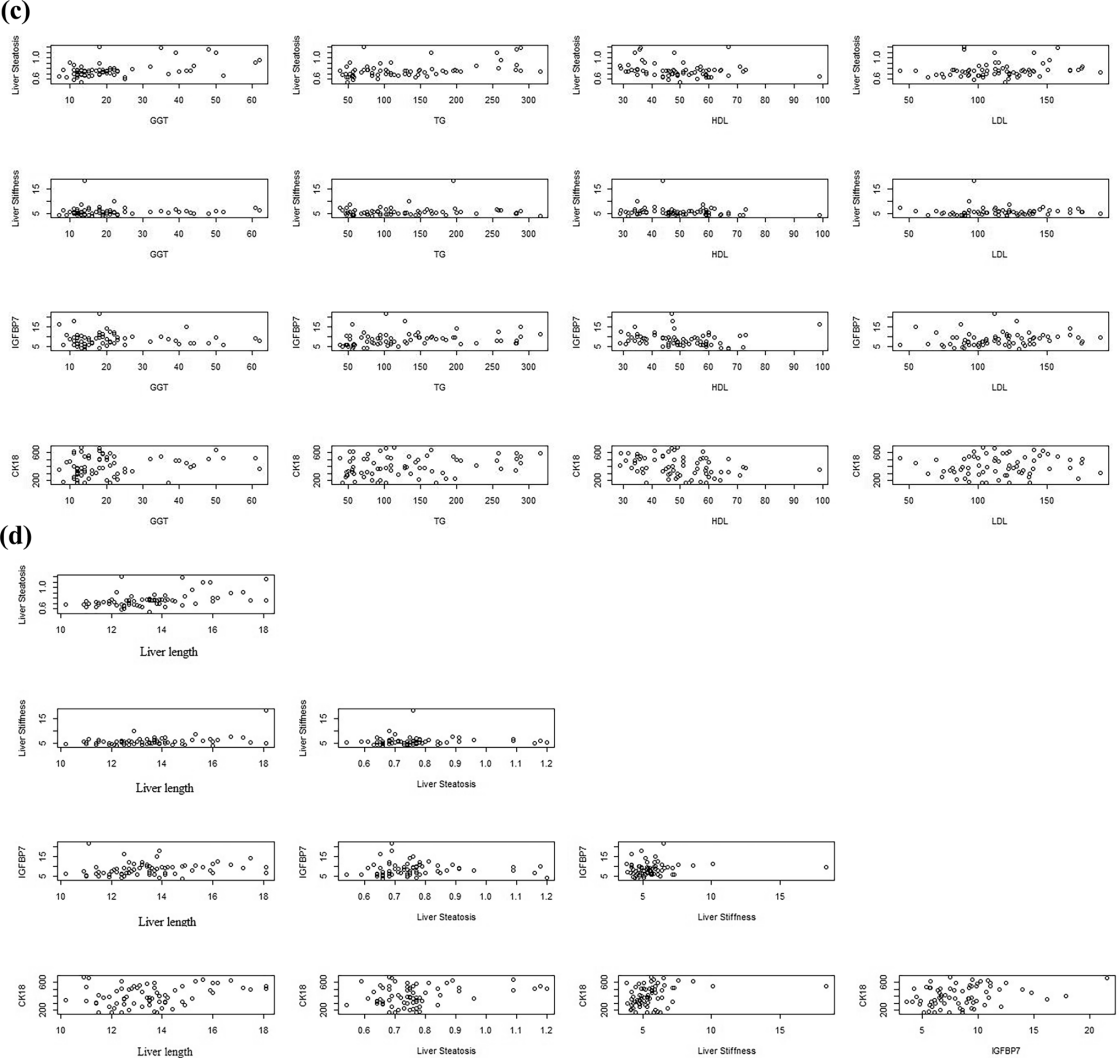
Correlation graphs of quantitative ultrasound techniques and biochemical markers

As shown in Table 2, liver steatosis was positively correlated with WC (r = 0.452, p < 0.001), BMI (r = 0.393, p = 0.001), body fat percentile (r = 0.359, p = 0.002), HOMA-IR (r = 0.357, p = 0.003), ALT (r = 0.413, p < 0.001), GGT (r = 0.346, p = 0.004), TG (r = 0.375, p < 0.001), and liver length (r = 0.517, p < 0.001) and negatively correlated with HDL (r = -0.254, p = 0.035). Liver stiffness was positively correlated with BMI (r = 0.328, p = 0.006), HbA1c (r = 0.384, p = 0.001), liver length (r = 0.275, p = 0.022), CK18 (r = 0.371, p = 0.002). IGFBP7 was positively correlated with WC (r = 0.268, p = 0.026), BMI (r = 0.262, p = 0.030), HOMA-IR (r = 0.295, p = 0.014), HbA1c (r = 0.386, p = 0.001), ALP (r = 0.425, p < 0.001), TG (r = 0.393, p = 0.001), LDL (r = 0.272, p = 0.108), liver length (r = 0.322, p = 0.007) and negatively correlated with HDL (r = -0.290, p = 0.016). CK18 was positively correlated with WC (r = 0.302, p = 0.012), HOMA-IR (r = 0.388, p = 0.001), HbA1c (r = 0.329, p = 0.006), ALT (r = 0.309, p = 0.010), ALP (r = 0.281, p = 0.019), TG (r = 0.256, p = 0.033), liver length (r = 0.256, p = 0.034), liver stiffness (r = 0.371, p = 0.002) and negatively correlated with HDL (r = -0.372, p = 0.002). In the acromegaly group, liver steatosis was negatively correlated with GH level (r = -0.456, p = 0.029). There was no correlation between CK18, IGFBP7, liver stiffness and GH, IGF-1 or IGF-1 ULN. Figure 2 presented the graphic displaying the correlation relationships.

**Table 2:** Correlation analysis between quantitative ultrasound parameters, serum biomarkers and clinical parameters

		WC	BMI	Body fat (%)	HOMA-IR	HbA1c	AST	ALT	ALP	GGT	TG	HDL	LDL	Liver length	Liver steatosis	Liver stiffness	IGFBP7
Liver steatosis	r	0.452	0.393	0.359	0.357	0.025	0.198	0.413	−0.150	0.346	0.375	−0.254	0.229	0.517			
	p	** < 0.001**	**0.001**	**0.002**	**0.003**	0.837	0.103	** < 0.001**	0.220	**0.004**	** < 0.001**	**0.035**	0.058	** < 0.001**			
Liver stiffness	r	0.151	0.328	0.039	0.072	0.384	0.036	0.180	0.097	0.220	0.052	−0.182	0.199	0.275	0.176		
	p	0.216	**0.006**	0.750	0.560	**0.001**	0.771	0.138	0.429	0.070	0.674	0.134	0.102	**0.022**	0.147		
IGFBP7	r	0.268	0.262	−0.010	0.295	0.386	0.075	0.215	0.425	0.103	0.393	−0.290	0.272	0.322	0.192	0.170	
	p	**0.026**	**0.030**	0.935	**0.014**	**0.001**	0.540	0.076	** < 0.001**	0.401	**0.001**	**0.016**	**0.024**	**0.007**	0.114	0.163	
CK18	r	0.302	0.208	−0.079	0.388	0.329	0.112	0.309	0.281	0.223	0.256	−0.372	0.108	0.256	0.207	0.371	0.294
	p	**0.012**	0.086	0.519	**0.001**	**0.006**	0.360	**0.010**	**0.019**	0.065	**0.033**	**0.002**	0.375	**0.034**	0.088	**0.002**	**0.014**

*WC* Waist circumference, *BMI* body mass index, *HOMA-IR* Homeostatic Model Assessment for Insulin Resistance, *HbA1c* glycosylated hemoglobin, *TG* triglyceride, *HDL* high-density lipoprotein cholesterol, *LDL* low-density lipoprotein cholesterol, *ALT* Alanine aminotransferase, *AST* Aspartate aminotransferase, *GGT* Gamma glutamyltransferase, *ALP* Alkaline phosphatase, *IGFBP7* Insulin like growth factor binding protein 7, *CK18* cytokeratin 18

Statistically significant p values were given in bold

As shown in Table 3, stepwise multiple linear regression analysis revealed that BMI (p = 0.008) and CK18 (p = 0.015) were independent risk factors for increased liver stiffness.

**Table 3:** Stepwise multivariate linear regression analysis for risk factors of increased liver stiffness

		SE	Beta	t	p	F	p	R^2^
Model 1	Constant	0.032		9.268	< 0.001	11.355	**0.001**	0.145
	BMI	0.001	−0.381	−3.370	**0.001**			
Model 2	Constant	0.031		9.851	< 0.001	9.208	** < 0.001**	0.218
	BMI	0.001	−0.306	−2.715	**0.008**			
	CK18	0.000	−0.281	−2.486	**0.015**			

Dependent Variable: 1 / Liver stiffness (kPa). Independent Variables: BMI, HOMA-IR, TG, HDL cholesterol, CK18, and IGFBP7

*SE* Standard error, *R*^*2*^ Explained variance, *BMI* body mass index, *HOMA-IR* Homeostatic Model Assessment for Insulin Resistance, *TG* triglyceride, *HDL* high-density lipoprotein, *CK18* Cytokeratin 18, *IGFBP7* Insulin like growth factor binding protein 7

Statistically significant p values were given in bold

## Discussion

In this study, we found that patients with acromegaly had higher liver stiffness, indicative of fibrosis, and lower hepatic steatosis. Furthermore, CK18 and IGFBP7were higher in the acromegaly group than in the control group. Finally, we showed that increased liver stiffness was associated with increased BMI and CK18.

Lipolysis, triggered by increased GH levels in acromegaly, leads to a decrease in both visceral and subcutaneous adipose tissue. However, while insulin resistance is expected to increase ectopic adiposity, as seen in metabolic syndrome, hepatic steatosis has been shown to decrease in acromegaly [26]. As far as we know, there is a limited amount of literature evaluating fatty liver with traditional ultrasound [27] and other QUS methods in acromegaly [28], however there is no evaluation with TAI. TAI is a quantitative ultrasonographic method that is easily applicable, has a high correlation with MRI-PDFF, and is highly reproducible [12, 29]. The median TAI was lower in the acromegaly group than in the control group and the number of cases classified as NAFLD was also lower in the acromegaly group than in the control group in our study, in accordance with the literature. Additionally, patients with acromegaly showed a negative correlation between TAI and GH, indicating that elevated GH levels are associated with low liver steatosis.

SWE is a highly reliable method for demonstrating tissue stiffness. Increased stiffness is associated with fibrosis in the liver unless there is an additional underlying disease [30]. Based on current guidelines, liver stiffness greater than 8 kPa on SWE is considered indicative of increased fibrosis [13]. Our study found that the acromegaly group had higher liver stiffness than the control group. Furthermore, increased fibrosis was observed in three of our cases using the fibrosis cut-off values for SWE recommended by current guidelines. However, this observation was indirect as a liver biopsy was not performed in our study. GH and IGF-I can induce fibrosis by increasing collagen synthesis and deposition outside of cellular overgrowth [31]. Additionally, increased GH has been stated to cause increased fibrosis with ductular reaction and proliferation in cholangiocytes [32]. The duration of exposure to elevated GH and IGF-1 in the cases included in our study is unknown. However, according to the literature, patients with acromegaly have an average delay in diagnosis of 5.5 years [33]. This prolonged period may potentially lead to the development of liver fibrosis.

In our study, IGFBP7 and CK18 were found to be higher in the acromegaly group than in the control group, supporting increased fibrosis and inflammation in the liver. IGFBPs are synthesized in the liver and are an essential part of the IGF system. IGFBPs promote cell proliferation and play a role in metabolic signalling by affecting glucose uptake, lipogenesis, glycogen storage, and the suppression of protein degradation and differentiation [34]. Circulating levels of IGFBP7 have been strongly associated with hepatic IGFBP7 expression as well as with steatosis and fibrosis stage in patients with NAFLD. It has been suggested that IGFBP7 may negatively affect the regulation of hepatic glucose and lipid metabolism secondary to insulin resistance due to its ability to bind directly to insulin receptors [15]. IGFBP7 has been shown to cause the activation of hepatic stellate cells, resulting in profibrotic activity [35]. IGFBP7 may be a good predictive method for the detection of mild fibrosis, although it has low sensitivity for APRI and FIB-4, the most widely accepted non-invasive fibrosis scores [16]. In our study, IGFBP7 was found to be higher in the acromegaly group than in the control group and showed high correlations with poor metabolic control parameters particularly HOMA-IR. However, no correlation was observed between the liver stiffness value and IGFBP7 levels. The insufficient number of cases may have overshadowed the demonstration of a possible relationship.

CK18 is a type I intermediate filament protein that is highly concentrated in hepatocytes and cholangiocytes, accounting for 5% of the total liver protein. The concentration of both intact CK18 and fragments resulting from caspase cleavage in the serum or plasma reflects the extent of necrotic damage to hepatocytes and/or apoptosis [36]. HCV-infected patients with T2D, CK18 levels were found to be well correlated with the fibrosis detected on liver biopsy. Furthermore, CK18 was detected as an independent predictor of liver fibrosis [19]. In our study, CK18 levels were higher in the acromegaly group compared to the control group. CK18 was positively correlated with WC, HOMA-IR, HbA1c and TG and negatively correlated with HDL, while there was no significant correlation with body fat. These results suggest that CK18 is affected by metabolic syndrome parameters. Additionally, CK18 showed a positive correlation with increased stiffness. Regression analysis revealed that CK18 was an independent risk factor along with BMI for increased stiffness. The liver may have responded to metabolic disruption by increasing fibrosis without steatosis in acromegaly, and fibrosis may lead to hepatic injury. In support of this argument, contrary to the classical sequential definition of NAFLD, it has been shown that liver fibrosis can develop independently of steatosis in some cases of metabolic syndrome [37]. In a population study that evaluated liver damage related to metabolic syndrome, 26% of the group had fatty liver disease, 7.5% had fibrosis associated with fatty liver disease, and 3.3% had fibrosis alone [37]. However in our study we observed that 13% of the acromegaly group had fibrosis independent of steatosis compared to no fibrosis in the control group. Since isolated fibrosis is not a common condition, we think that the significance of the findings should not be overlooked. In the literature, despite hyperinsulinemia and insulin resistance, low cardiac lipid accumulation was found in active acromegaly similar to low hepatic lipid accumulations [38]. Additionally, common cardiac problems in acromegaly, such as biventricular hypertrophy, diastolic and systolic dysfunction and valve disease, are known to occur as a result of myocardial fibrosis and inflammation [39, 40]. We think that increased liver stiffness in newly diagnosed acromegaly patients may be a result of early liver damage other than ectopic lipid accumulation beyond simple fibrosis and that increased CK18 may be an effective early biomarker for detecting this damage.

Our study had some limitations. Although our small number of patients may be considered a limitation, the inclusion of only newly diagnosed acromegaly patients is important because it prevents heterogeneity that may occur in the treatment group. The exclusion of diabetic patients from the control group may be seen as a limitation of the study. However, it is noteworthy that the control group still had a high prevalence of metabolic syndrome (28%) and NAFLD (52%) even without diabetic patients. Additionally, the acromegaly group less liver steatosis than the control group, despite the absence of diabetics in the control group. Furthermore, our study was presented as a cross-sectional study, not a prospective study. This prevented us from showing possible changes in liver stiffness after hormonal control was achieved. According to the literature, liver stiffness has been evaluated in a limited number of studies in acromegaly but prospective follow-up was not performed [5] [6]. Therefore, it is thought that reassessing the study group with both imaging and biochemical markers after disease control will aid in interpreting the current findings. Finally, the presence of fibrosis was not confirmed by biopsy in our study. Although CK18 and IGFBP7 are thought to be associated with increased fibrosis in the liver, further studies with more comprehensive histopathological examination are needed to confirm this.

To our knowledge, our study is the first to investigate IGFBP7 and CK18, thought to be markers of increased hepatic steatosis and fibrosis, in patients with acromegaly. We also used TAI for the first time to evaluate fatty liver in acromegaly. In conclusion, our results indicated increased liver fibrosis along with decreased liver steatosis and serum CK18 might be a potential marker of increased liver fibrosis in acromegaly.

## Data Availability

The datasets generated during and/or analysed during the current study are available from the corresponding author on reasonable request.
